# The Ethics of Motivational Neuro-Doping in Sport: Praiseworthiness and Prizeworthiness

**DOI:** 10.1007/s12152-020-09445-5

**Published:** 2020-07-23

**Authors:** Bowman-Smart, Hilary, Savulescu, Julian

**Affiliations:** 1 Biomedical Ethics Research Group, Murdoch Children’s Research Institute 50, Rd Parkville VIC 3052, Flemington, Victoria 3052 Australia; 2 Department of Paediatrics, University of Melbourne, Parkville, Victoria 3010 Australia; 3 Uehiro Centre for Practical Ethics, University of Oxford, St Ebbes St, Oxford, OX1 1PT UK

**Keywords:** Sport, Doping, Neurodoping, Motivational enhancement, Praiseworthiness

## Abstract

Motivational enhancement in sport – a form of ‘neuro-doping’ – can help athletes attain greater achievements in sport. A key question is whether or not that athlete deserves that achievement. We distinguish three concepts – praiseworthiness (whether the athlete deserves praise), prizeworthiness (whether the athlete deserves the prize), and admiration (pure admiration at the performance) – which are closely related. However, in sport, they can come apart. The most praiseworthy athlete may not be the most prizeworthy, and so on. Using a model of praiseworthiness as costly commitment to a valuable end, and situating prizeworthiness within the boundaries of the sport, we argue that motivational enhancement in some cases can be compatible with desert.

Achieving in elite competitive sport relies on many factors. There is, of course, the matter of natural physical abilities and capacities that are essential to reach the elite level. However, equally important is the drive the athlete has towards reaching their goal. The field of sport psychology has dedicated extensive amounts of research to the matter of motivation in sport.^1^. If there are two equally matched athletes, it is the one who is motivated to train harder and longer that will have the edge. Elite athletes must combine physical prowess with psychological resolve to reach the top of their field. In addition, the exercise of various mental capacities (strategy, determination, drive, etc) during competition are essential.

Enhancement in sport is frequently discussed in the context of enhancement of physical capabilities, using interventions such as anabolic steroids.^2^. However, attention has turned to enhancing the cognitive abilities and motivation of athletes. This may be achieved through methods such as psychological and behavioural interventions.^3^. This approach is generally seen as acceptable and not a form of ‘doping’.

Motivation is a key component of an athlete’s performance. Cognitively enhancing an athlete’s ability to achieve is one thing – that is, enhancing their ability to be *able* to do it. Equally important, but less discussed, is the enhancement of the athlete’s motivation to engage in training and make steps towards their goal – whether or not they *will* do it. This can be referred to as the ‘can’t/won’t’ distinction.^4^. What matters is not just the capabilities of the athletes, but how they develop and use them. The latter kind of enhancement is ‘motivational enhancement’.

Motivation “is about wanting to make the effort necessary to do a task”.^5^. Motivation is best thought of as a *state*, which is a disposition or inclination towards performing a particular action. Effort, on the other hand, is something that is exerted through *acting*, and the exertion of effort produces aversive psychological features.^6^. Motivation alone is not sufficient to achieve an end. Someone may be very motivated to win an athletic competition, but unless they exert the effort to pursue that end, they will not achieve it. However, motivation and effort are closely related. If someone is highly motivated to perform an action, they will have to expend less effort to do it: although the action might require the same amount of energy, expending this energy will feel much less effortful and therefore less aversive. Conversely, if someone has very little motivation, the effort they will have to expend will be much higher and will feel more aversive.

There exists a range of methods for enhancing various aspects of performance, some of which achieve their effects by directly or indirectly modulating brain activity or function. Such techniques, where they confer performance benefits, are instances of ‘neuro-doping’. Existing techniques include taking pharmaceutical drugs such as Modafinil or Ritalin.^5^. They may also include the use of trans-cranial direct current stimulation (tDCS),^7^, which involves stimulation of certain areas of the brain. Neuro-doping can be used to increase cognitive capacities, motor skills, and neural entrainment, helping athletes achieve better performance.^8–10^. Neuro-doping may also be used in cognitive sports, such as chess.^11^.

Motivational enhancement, as we will use the term, is the use of pharmaceuticals or technology to increase someone’s motivation to complete a particular task or action. Neuro-doping also has possibilities for motivational enhancement. tDCS can reduce the propensity to mind-wander^12^ and can enhance endurance in the sporting context.^13^. It may also enhance the mood of elite athletes, but not necessarily their performance.^14^. tDCS as a technique is still in the experimental stage for enhancement, and there is conflicting evidence as to its effects. However, it is important to discuss its potential impact on sport and the principles we would use when considering how to govern these technologies.

Some, such as Kjærsgaard, suggest that the widespread use of stimulants such as Ritalin and Adderall is due at least in part to their motivational effect.^5^. Although these drugs are often described as ‘cognitive enhancers’, it may be that they are having more of an impact on the motivation, concentration and/or attention of the user. This view that their usefulness stems from their capacity to increase motivation, feelings of energy and attention performance, rather than their impact on cognitive performance (which may in fact be weak), has some support.^4, 15–17^. Stimulants such as Adderall may indeed have a deleterious impact on functions such as working memory.^16^. However, there is stronger evidence that these drugs increase subjective feelings of their capability, which may in turn increase motivation, even if these feelings do not correlate with objective measures.^18, 19^. Ritalin reduces the cost-to-benefit ratio of cognitive work, and thus boosts willingness to do the task.^20^. They can also increase task enjoyment, which again reduces the effort required to complete the task.^21^. Amphetamines can increase the amount of willingness to exert effort in order to get a reward.^22^.

There are two different types of motivation to consider.^23^. The first is intrinsic motivation, where the agent finds pursuing the task inherently rewarding or enjoyable (for example, they enjoy training for the sport). As described above, certain kinds of drugs can increase task enjoyment, which is related to intrinsic motivation. The second kind of motivation is extrinsic motivation, where the agent pursues the task in order to achieve a separate outcome (for example, the agent trains for the sport, despite not enjoying it greatly, in anticipation for the reward of winning/first place). This type of motivation may require more self-regulation. Pursuing a goal when driven by extrinsic motivation may require more effort than when driven by intrinsic motivation. Motivational enhancement may impact both kinds of motivation; Modafinil may increase subjective task enjoyment (intrinsic motivation), and amphetamine-based substances may increase willingness to endure effort to receive a reward (extrinsic motivation).^6^.^1^

Motivational *doping* would be the use of motivational enhancement in a context such as elite sport. If an athlete used motivational doping, they could train harder and longer as the effort required to do so is less. Motivational doping is likely to give most benefits over time, such as in the context of training for competition. When an athlete is choosing to train, having the motivation to train an extra hour each day may make the difference between being on the podium or not. Motivational doping could also enhance the athlete’s performance on the day by, for example, increasing feelings of energy.

Here, we are not arguing straightforwardly in favour of introducing motivational doping in sport, but instead aim to provide an architecture for discussion, and identify relevant considerations pertaining to its use. We argue that motivational doping does not necessarily reduce praiseworthiness or what we will call ‘prizeworthiness’ in elite athletes.

## Praiseworthiness, Prizeworthiness and Admiration

Some perspectives view achievements, such as those in elite sport, that are made with the help of enhancement to be ‘undeserved’ because the enhancement allows the agent to avoid effort.^24^ On such views, the agent may be less praiseworthy.^25^ Here it is important to distinguish several key related concepts – which are praiseworthiness, prizeworthiness, and admiration. These are different things, and they do not always go together. Some of the difficulty with attaining clarity in discussion of enhancement in sport is that these concepts are sometimes conflated, or prize- and praiseworthiness are assumed to co-instantiate. However, the most praiseworthy athlete may not be the one that gives the most admirable performance, nor the one that deserves first place (i.e. is the most prizeworthy). Someone who has overcome great challenges in order to complete a marathon (for example, by overcoming collapse from exhaustion and dehydration) may be the most praiseworthy, but this does not mean they have run the fastest race or automatically deserve first place. In sport, these concepts can come apart.

Let us begin by defining praiseworthiness. The model proposed by Maslen et al. suggests that praiseworthiness is dependent on the agent’s ‘costly commitment’ to a particular valuable end. According to this view, the morally relevant aspects of costly commitment are (i) the voluntariness of the committed pursuit of the end, (ii) the costliness of the committed pursuit of the end, (iii) the value of the end being pursued, and (iv) the strength of the agent’s commitment to the end.^6^

This means that the agent needs to choose to engage in the pursuit of a particular valuable end, and incur costs and/or demonstrate commitment in doing so. One example of a cost that can be incurred is the exertion of effort, which produces aversive psychological features. Similarly, an agent can demonstrate commitment by, for example, pursuing this particular end above other ends – priority-setting. In addition, the value of the end is important. An agent is not praiseworthy for pursuing a valueless end (such as counting blades of grass), and the value of the end is not dependent solely on how the *agent* values it, but its objective value. The importance of the value of the end is also relative to the agent’s capacity to achieve it. For example, even if it happened to be very valuable to count all the blades of grass, if the agent has no capacity to achieve it, then it is a waste of time to attempt to do so and thus the agent is not praiseworthy.

We extend this model in the context of sport, and add two further conditions. These conditions must be added because sport is an activity where it is not enough to simply reach a particular valuable end. Those valuable ends (e.g. winning a race) must be reached in a specific way – that is, within the spirit of the sport. Indeed, reaching these ends within a specific way is an essential component of the construct of sport.

Firstly, the agent’s role in the actual performance, their *doing* something – their agential contribution - must not be diminished so far that it is outside the parameters of the sport. As we will discuss, these parameters are subject to negotiation; some sports require more agential contribution than others. Secondly, the costs incurred must be directly related to their agential contribution to the performance. How to determine whether or not the costs are related depends again on the particular sport. In order to be praiseworthy specifically for their achievement in a sport as an end in itself, an athlete must fulfil these two additional conditions.

Furthermore, as stated, we must distinguish praiseworthiness from similar concepts such as admiration and prizeworthiness. Admiration is the pure appreciation or wonderment at the product itself (which is, in this case, the sporting performance or achievement). We can admire these products even when the agent is not praiseworthy. For example, I could watch a cheetah or a robot run very fast and admire this performance greatly, but that does not necessarily mean I accord the cheetah or the robot any praiseworthiness. Admiration and praiseworthiness frequently come together, but they can come apart.

Similarly, as we will discuss further, praiseworthiness and prizeworthiness are not necessarily one and the same. It is important to distinguish these two concepts when we are considering what athletes ‘deserve’. Deserving *first place* (prizeworthiness) is not determined by the level of praise we should attribute to the athlete. Someone who has overcome an extreme amount of obstacles, incurred enormous costs, and demonstrated very strong commitment may come fortieth in the marathon, and they may be the most praiseworthy in the race. However, they do not therefore deserve first place. In order to deserve first place, the athlete must fulfil the relevant victory conditions (in this case complete the course in the shortest time), within the constraints of any rules set for the competition (i.e. not by rollerblading her way to victory). Prizeworthiness also does not necessitate an admirable performance – for example, a football team may play a very defensive game and win that way, or we can refer to the case of Australian skater Steven Bradbury winning at the 2002 Winter Olympics by virtue of all of his competitors falling over^26^^.^^2^First place is not just a prize for effort, it is a prize for a certain kind of performance. The criteria for deserving first place – for being prizeworthy - are not the same as the criteria for being praiseworthy.

The victory conditions are typically set by the excellences which the activity is meant to capture or exhibit, and the level of agential contribution that is typically required of the sport. These factors could be described as capturing the ‘spirit of the sport’. In addition, there will also be an element of luck. As we will discuss, determining exactly how to create victory conditions that capture the ‘spirit of the sport’ is an ongoing negotiation within sporting codes. Although we argue that being praiseworthy (or, of course, prizeworthy) within the context of a sporting event is dependent on operating within the rules, this does not mean that we regard the rules as fixed. The rules of the sport can be adjusted, with the goal in doing so being to preserve the ‘spirit of the sport’. We argue that motivational doping is compatible with those negotiations and should not be dismissed out-of-hand as being necessarily ‘incompatible’ with the rules.

## The Athlete-as-Agent

Although they are different, both praiseworthiness and prizeworthiness require that the athlete contribute as an agent to the performance, and contribute in a particular way. As with many other arenas in life, sporting achievements must be done within certain parameters to warrant praiseworthiness or prizeworthiness for the achievement as an end in itself. Here, we will discuss the necessity of agential contribution and how this can be negotiated. We must then consider how motivational doping may affect the agential contribution.

One of the key aspects of the nature of most sports is that it is an activity done by agents. That is, the agent (we will focus on human agents) is exerting some effort to perform or intentionally achieve something. Achievement in sport involves costs, such as effort; technology often affects the degree and nature of effort. If technology reduces the athlete’s effort by too great a degree, this must affect how we view the athlete’s achievement. This is because the effort is displaced from the athlete, who is meant to be the agent, and onto other agents – the creators of the technology that have enabled the performance. For example, if we consider a competition between robots, the agent who exerts the effort (and is thus praiseworthy) is the person who *built* the robot. We do not expect human athletes to be robots. We expect them to be agents themselves, and that they need to be in order to be praiseworthy. One of the important parts of the nature of sport is the costly commitment of the athlete-as-agent.

Sporting achievements are dependent upon the parameters of the sport. For example, if you attempted to undertake a marathon wearing rollerblades,^2^ you would not be permitted to do so, because the nature of the sport is to demonstrate the ability to *run* long distances, not just cover a long distance by any means. If you completed a marathon wearing rollerblades, you may be praiseworthy in some way (it is presumably no small task to rollerblade 42 km), but you would not deserve a prize for coming first, or even praise for completing the marathon per se. This is because you must achieve the end within certain parameters, and using rollerblades is outside these parameters.

The boundaries of these parameters are not, however, fixed. They may be negotiated and change with the development of new technologies. We may say that one technology reduces the agential contribution required *too much* and thus is no longer within the nature of the sport. For example, there has been significant recent controversy over the use of shoes such as Nike’s Vaporfly in long-distance running, as they can improve an athlete’s performance by 4%.^27^ After much debate, the shoes will be allowed under new rules.^28^ This kind of negotiation of rules and standards to determine what is within the ‘nature’ of the sport and what is not, is inevitable with any new development. The nature of the sport is not some fixed construct; it is a social construct that is modifiable. Motivational doping is subject to the same debate.

In order for technology to be compatible with the nature of the sport, while upholding the athlete as the agent worthy of praise, there needs to be a minimum level of agential contribution on the behalf of the athlete. If we reduce this too much, then it is not the athlete-as-agent that has produced the achievement. It is likely that in general, there needs to be some level of involvement of human agency that is a minimum, and then there is a large grey zone where sports can involve more or less human agency. This ‘grey zone of agency’ is where the negotiation of how to integrate technology into the parameters of the sport occurs. Some sports may accept more, and some sports may accept less. We argue that motivational doping is compatible with negotiation in this grey zone of agency.

For some sports, the agential contribution of the athlete may be extensively modified or supported by other agents. Many sports use technology that removes or shifts agency from the athlete on the field. For example, motor racing requires a whole team of engineers and mechanics who are not on the track to win the race; the viewer knows that it is not just Kimi Räikkönen who has won the race, it is the Ferrari *team* who designed and built the winning car. In this case, the viewer knows that there are agents beyond the driver who also deserve praise and recognition for achieving first place. The level of visibility of these agents may vary between different sports. Any sport where a piece of equipment or an animal (e.g. horse racing) significantly contributes to performance, we accept the role of agents off the field (e.g., we praise the horse trainer) or even perhaps non-human agents (e.g. the horse – in this case, the jockey and the horse form a team). Here, when we are discussing motivational doping, we are primarily considering the motivational enhancement of athletes on the field (whether as teams or individuals) – i.e. the athlete-as-agent.

The level of cost (such as effort) that it is expected for the athlete-as-agent to expend can vary significantly between sports. For example, we can consider the various approaches to elite cycling. The mean speed in the Tour de France in 2008 was roughly double what it was in 1892; much of this can be attributed to the technological improvement of bicycles, particularly in terms of their weight.^29^ Bicycle development has been enormous since the advent of cycling competitions, in all arenas. As with the Nike Vaporflys, sport bodies such as the Union Cycliste Internationale (UCI) often negotiate with what technological developments they will ban and what others they will accept; for example, the UCI initially banned the pneumatic tyre and the derailleur after their invention, but these bans were then repealed and these technologies are now common.^30^ In many ways, achievement in the various varieties of cycling comes down to the right bike as well as the right rider.

Some kinds of cycling try to emphasise the abilities of the rider by removing the impetus for technological development as much as possible. Keirin cycling, a sport that originated in Japan, requires that all riders use very similar bicycles built to a strict standard. All parts must be stamped and approved by the governing body and there are a small number of approved bicycle builders. Bicycles used in Keirin racing are made from steel, as opposed to other cycling competitions (e.g. the Tour de France) where carbon fibre is the norm. This is to reduce any difference in advantage conferred by bicycle technology, as well as the contribution of technology to racing achievements, and instead emphasise the abilities of the cyclist in the competition.

However, for all sports, there is some level where transferring the agential force from the athlete to the providers of the technology unacceptably transforms the nature of the sport and therefore the praiseworthiness of the athlete. There may be some minimal level of agential contribution that is required for an athletic performance to be within the bounds of the nature of the sport, and thus praiseworthy. The key question is whether motivational doping crosses this line. In some extreme cases, motivational doping may do so. It would affect praise in pursuit of valuable ends when the reduction of cost is too great, it reduces the necessity of the athlete to demonstrate strength of commitment, or it renders the agent passive in the pursuit of the athletic achievement. Motivational doping, in the forms in which it is currently available, does not necessarily do this.

An example of extreme motivational doping that *would* do this would be something like a brain implant that completely removes the choice and/or cost from the agent. For example, this implant may lead them to compulsively train as much as possible, which removes the voluntariness of the agent. Therefore, the agent could not be praiseworthy. The implant could also make training extremely pleasurable and remove the aversive psychological features associated with effort. This would reduce the costs associated with the pursuit of the end so greatly that the performance is no longer within the nature of the sport and thus the athlete-as-agent’s performance is no longer praiseworthy.

However, current methods of motivational doping (such as tDCS or Ritalin) may modify the agential contribution, but they do not do so any more than a spectacularly good trainer or a top-of-the-range bike. Motivational doping should be subject to the same kinds of negotiation as any other technologies in sport that modify agential contribution.

Whether or not the rules should change depends on how we weigh the agential contribution in the context of the sport. It is trivial to say that if you follow the rules, you deserve the prize. The question is more what those rules should be. As we have outlined, rules are already adjusted to preserve the spirit of the sport. We argue that motivational doping is no less eligible to be part of those negotiations than other technologies that modify performance.

## Motivational Doping and Praiseworthiness

Another one of the key morally relevant aspects of praiseworthiness would be the costliness of the committed pursuit of the end. Motivational doping would reduce the effort required to train. Effort is marked by aversive psychological features, and is a form of cost. Therefore, motivational doping could reduce the cost of the pursuit of first place. However, motivational doping may incur other costs that balance out this reduction in costs. For example, taking Ritalin may affect your sleep or have other physiological side-effects.^31^ Another cost may be taking the risk that there are as-yet unknown side effects. It is important to note that if motivational doping incurred great costs to the point that the techniques were dangerous, this would be a reason for it not to be allowed in sport. This reason is a pragmatic one, due to concern for the athlete’s safety and not the praiseworthiness of their performance.

In addition, motivational doping could result in the agent spending much more time training that they could spend on the pursuit of other ends valuable to the agent (an opportunity cost). Thus, when the agent is undergoing motivational doping, the agent may well be choosing to incur some costs (missing out on other opportunities) rather than others (aversive exertion of effort). How this will affect the agent’s praiseworthiness will depend on the net balance of costs. For example, if the motivational doping method incurred *no* cost (such as, possibly, tDCS), this may make the agent less praiseworthy than if they had used a motivational doping method that incurred some cost (e.g. as stated, inability to engage in other ends valuable to the agent).

However, as we have stated, it is important that these costs are directly related to the agential contribution to the performance. Once can incur extreme life costs in order to reach the valuable end that are not related to training, preparation, or the performance itself. For example, one may make a deal with the devil to win a race at the Olympics in exchange for an eternity in hell.^3^ This incurs great cost. However, it is not related to the agential contribution of the athlete to the performance. In fact, in this extreme example, it entirely removes the agential contribution to the physical performance. As we have extensively discussed, if we entirely take away the agential contribution to the performance itself, this affects praiseworthiness in the context of that specific sport performance. Just as rollerblading to victory in a running race would be neither praiseworthy or prizeworthy, nor would it become so even if the purchase of the roller blades bankrupted the person*.*

Ends such as sporting achievements have defined parameters in terms of how they can be reached. In order to be praiseworthy for a sporting achievement, you must achieve that end by certain means – incurring certain kinds of costs. The costs you incur must be specifically related to the agential contribution you make to the performance. If you do not achieve that end by those particular means, then you may be praiseworthy for something, but you are not praiseworthy for achieving the sporting end. For example, if you bribe your way to win a boxing match in order to donate the prize money to charity, then you may be praiseworthy for achieving the valuable end of donating the prize money to charity, but you are not praiseworthy for winning the boxing match. The means need to be a certain kind related to the agential contribution of the athlete to the performance.

The key problem arises when we try to determine how cost relates to agential contribution. Here, we must again negotiate this concept depending upon the sport and the level of agential contribution it requires from the athlete. For example, in rugby, costs related to the agential contribution to the competition may include effort exerted in running, weightlifting, team-building exercises, strategy sessions, and so on and so forth. Costs incurred that are outside the parameters of the sport include things such as monetary loss from bribing the referee, or selling your soul to the devil. What we must think about is whether the costs incurred from motivational doping are sufficiently related to the agential contribution. Motivational doping, in the forms we have proposed, has an *effect* akin to a trainer who highly motivates you and pushes you to train further. This would similarly result in costs such as the opportunity cost described above. In addition, athletes frequently do things in the course of training that result in some risk to their health, and we regard those as valid costs. We believe there is a strong case to be made that the costs of motivational doping can be related to the agential contribution of the athlete in a way that making a deal with the devil does not.

We now move on to assessing the impact of motivational doping on the other aspects of praiseworthiness, from the model on which praiseworthiness is a function of an agent’s voluntary, costly commitment to a valuable end. Let us first examine the relationship between motivational doping and voluntariness. In the case of elite athletes, it is likely that they have made an active choice to engage in the sport of their choice. Assuming that voluntariness is not being impeded by other means (e.g. coercion), it is not clear how motivational doping would affect voluntariness. Firstly, the impact of the technologies described is not so great that it would remove the capacity of the agent to voluntarily make a choice to pursue a particular end. Secondly, motivational doping would not direct motivation towards one particular end, it would just produce increased motivation as a general disposition; the agent must choose where to direct that disposition. Therefore, motivational doping would not appear to impact praiseworthiness in relation to this aspect.

It is important that the standard for voluntariness is not high. For example, let us consider the case of the adult who pressures a child to achieve in sporting activities. Parents play an important role in children’s participation in sport, and that role can be positive (supportive of their child’s agency) or negative (pressuring or forcing them to engage in a sporting activity, such as training).^32^ Similarly, other adults involved such as coaches can engage in negative behaviour, to the extent of committing severe emotional abuse of young elite athletes.^33, 34^ There is also the more extreme case of child athletes whose intense training regimes are driven by the state.^35^ In many cases, the child’s decision-making will be shaped and inextricably linked with the goals of the adults around them. Many of these young athletes go on to perform incredible feats and achieve significant wins in sporting championships. In many cases, these athletes would not have chosen and stuck to the paths they have taken without the influence of the adults around them. However, generally, their choices retain sufficient voluntariness to retain praiseworthiness. Although we may think it bad for the child’s wellbeing, we generally accept the influence of parents’ ambitions on children’s sporting careers in terms of the child’s praiseworthiness. It is unclear how motivational doping could be worse than this. As stated, motivational doping is not forcing the child to pursue the particular end, as the parent is doing. We will not continue to delve too deeply into the concept of what it means for an action to be ‘voluntary’, but it is important to say that particularly in the context of sport, where pressure is high, the standard of voluntariness for the purpose of praiseworthiness should not be so high as to deny many athletes praise.

However, the cost to the agent of pursuing the end is not the singularly determinative factor. It is also important to discuss another key aspect, which is the strength of commitment. Taking a motivational enhancer can demonstrate strength of commitment and priority-setting. The agent may strategically reduce costs by using a motivational enhancer, and this can indicate strength of commitment. Motivational doping could be an important part of achieving a valuable end, and the steps the agent takes to do so demonstrates that they are prioritising this valuable end over others. They change and adjust their plans according to the methods they employ.

The value of the end is also important when we look at praiseworthiness. It is important for an end to be valuable in order for you to be praiseworthy. For example, you are not praiseworthy for incurring great costs when the end is not valuable enough e.g. swimming in dangerous waters to save a child vs swimming in dangerous waters to save someone’s lost hat (not praiseworthy). The costs here are not gratuitous in the sense that they need not be incurred to reach the end (they do, there is no other way to get the hat), but they are gratuitous in the sense that they are completely disproportional to the value of the end.

The value of the end must also be relative to the agent’s capacity to reach that end. For example, if Usain Bolt were to join an amateur 100 m race and won, that end would not be very difficult for him to achieve, and thus he would not be particularly praiseworthy for simply winning. However, it would be very valuable in the context of the amateur sprinters’ ability to achieve that end. Conversely, if the amateur sprinter were somehow able to join the Olympic 100 m race, the related end (winning) would be very valuable but the athlete would have no capacity to achieve it. Therefore, they would not be as praiseworthy as it would effectively be a waste of time as the goal cannot be achieved. Agents are praiseworthy for undergoing maximal costly commitment to pursue a valuable end that it is within their capacity to achieve. To some degree, this may admittedly depend on luck (if someone is 5 ft tall it is unlikely they will ever to be able to make it in professional basketball). Natural capacities are to some degree dependent on luck.

It is important that the value of sport in the context of praiseworthiness does not depend solely on the particular athlete’s perspective of that value. For example, an athlete might not view second place at the Olympics as particularly valuable, but achieving second place would still likely have some objective value. The value of sport is dependent upon societal context. For example, a society of mole people may not value achieving a 100 m sprint, but it does have value within the context of our human society. Sport does not have objective value in the same way as saving someone’s life, but this does not mean it is relative solely to the individual’s valuation of their achievement. This is because sport is a collective endeavour and has social significance. The rules are set collectively, which is why if the individual breaks them their achievements have little value within the context of the particular sport.

As with other parts of the costly commitment model, there is a relationship between the variables. There is a relationship between cost and value. If something is very difficult for humans in general to achieve (i.e. it has high costs to the average person), it is often seen as more valuable. Part of the reason that winning an Olympic medal is more valuable than winning the local community football game is because the costs to get an Olympic medal are generally much higher. Therefore, there may be concern that reducing the costs required to obtain an Olympic medal, through motivational doping, makes achieving it less valuable. We have argued this is not necessarily so.

## The Value of Ends in Sport

Much of the literature around motivational enhancement refers to the value of ends in terms of what we will refer to as their non-positional value. For example, a surgeon may be more motivated to save someone’s life, and this has non-positional value. A life has been saved, and the achievement of this end did not require defeating anyone else in its pursuit. Amateur sports, too, often have ends that do not derive from winning against another agent (for example, because you are having fun and getting fit). However, while some ends in elite sport have non-positional value in themselves (e.g. mastering a particular manoeuvre in gymnastics), competitive elite sport has ends that primarily have *positional* value. For example, coming first in a 100 m sprint is relative to other agents’ attempted pursuit of that same end. If there were no other agents attempting to pursue that end, the end of ‘coming first’ or ‘winning the race’ would be meaningless.

It is important to distinguish these kinds of ends when considering motivational doping in sport. For example, there may be five competitors in a marathon. All five of the competitors complete the course. This is a non-positional end; you can run the marathon by yourself, with nobody else in the race, and still obtain this end. Other non-positional ends that we might consider might be completing the marathon in a certain amount of time (e.g. the minimum needed to qualify for the Olympics). Achieving these ends is not dependent upon the presence of other agents pursuing the same end.

The five competitors may be more or less praiseworthy depending on the costliness and strength of their commitment to that end. If one competitor naturally requires less training and deprioritises their pursuit of that end, and thus incurs less cost and demonstrates less strength of commitment, they may be less praiseworthy. Therefore, when considering distributing praiseworthiness amongst the competitors in pursuit of this particular end (finishing the recreational marathon) which they all achieve, the praiseworthiness of this end need not necessarily correspond to the competitor’s *position* in the race (i.e. the first place competitor is not necessarily the most praiseworthy).

However, only one competitor wins the race by coming first. This praiseworthiness of attaining this end is relative to other agents’ pursuit of that end and their performance in pursuit of that end. The competitor who came first is the only one who successfully achieved the valuable end of coming first. This concept of the ends in sport is also particularly relevant to sports where ‘winning’ comes not through comparison but through conflict (e.g. boxing, or arguably a football match) and the non-positional end (e.g. making it all the way through the boxing or football match) is usually relatively meaningless (although not always – a team may know they have no chance of winning, and thus their end is to achieve a certain number of goals, which can be done strategically in tournaments). Succeeding in the pursuit of the end of ‘winning’ or ‘coming first’ is something only one competitor or team can do.

However, the costly commitment requirement for praise does not mean that the agent has to obtain the end, merely that they have to pursue it. They deserve praise not just for (i) completing the course of the marathon, but also (ii) pursuing the first place in the marathon. Therefore, the competitors who attempted to but did not come first may indeed deserve praise, and the amount of praise they deserve may not necessarily track with the place they achieved in the race or the tournament. Again, the first-place competitor may not be the most praiseworthy in the race.

## Purity of Motivation

Another key aspect related to motivational doping in sport is the ‘purity’ of motivation that affects praiseworthiness. For example, an athlete may enter the race and pursue first place solely because they want the prize money in order to gamble it; thus, this makes the pursuit of first place an instrumental end. One might argue that this makes that athlete less praiseworthy than an athlete who is doing it purely for the ‘love of the sport’. Other acceptable motivations might include, for example, seeking to fulfil regional or national pride, or because it was your dying mother’s last wish. In all these examples, the athlete may be considered to be praiseworthy when they are motivated by good reasons rather than bad reasons.

Athletes can pursue sporting achievement for instrumental or intrinsic ends, as well as good and bad reasons. Reasons generally considered ‘bad’ (e.g. for gambling) would mostly be for the pursuit of instrumental ends. ‘Good’ reasons can also be for instrumental ends – for example, to make your mother happy. What impact does this have on the praiseworthiness of an athlete? It may be that if you are motivated by your mother’s happiness, you are more praiseworthy as a child, but less praiseworthy as an athlete.

According to this view, motivational doping may make the motivation to achieve ‘impure’, because the athlete’s motivation is artificially generated, and empty or hollow. This kind of motivation is not ‘good’ and this affects praiseworthiness. However, motivational doping can increase intrinsic motivation, such as the ‘love of the sport’, which is a ‘good’ reason. It can also enhance the exertion of effort to achieve a reward, such as money for gambling. Motivational doping may merely increase the fervour of reasons that are already there, whatever their level of goodness. When considering this, we may be straying into discussions of enhancement and authenticity of self, which is beyond the scope of what we can discuss here.

It is important to take a realistic view of motivation. Athletes can be motivated by many things, and picking apart motivations would be extremely difficult. An athlete may seek to win both for ‘selfish’ and ‘non-selfish’ reasons. Who is to say what makes a motivation impure? In addition, the view of praiseworthiness as costly commitment says nothing about how the motivation is generated apart from its relationship to the cost and strength of commitment. An agent is praiseworthy when they pursue a valuable end regardless of whether they are pursuing the end for itself, or as a subsidiary in the pursuit of some other end.

## Conclusion

Motivational doping in sport poses unique questions compared to other forms of enhancement. Where other kinds of enhancement aim to improve an athlete’s physical and cognitive capacities, motivational doping aims to improve an athlete’s will. Motivational doping may make it easier for athletes to engage in arduous training regimes, but they are nonetheless arduous.

According to the costly commitment model, motivational doping can still result in praiseworthy athletic performances. However, it may be that some extreme forms of motivational doping are not acceptable. These extreme forms would include if they removed the voluntariness of the athlete, the costs are reduced too greatly, they reduce the strength of commitment required, or they remove the agential contribution of the athlete.

The introduction of new technologies and processes into sport must be negotiated. There is no reason that motivational doping could not similarly be subject to such negotiations. There can be a place for motivational doping in elite sport and, if motivational doping were to be accepted, athletes would still be prizeworthy, regardless of whether their degree of praiseworthiness is affected.

## Footnotes

1 An anonymous reviewer has raised the possibility that prolonged use of motivational enhancement may undermine intrinsic motivation. This is a possibility that would need to be explored and, if true, a cost that may need to be considered when evaluating the use of motivational enhancement.

2 Thank you to an anonymous reviewer for this point

3 Thank you to an anonymous reviewer for this example
